# CDC COVID-19 healthcare infection prevention and control assistance to health departments, January 2020–December 2021

**DOI:** 10.1017/ash.2022.122

**Published:** 2022-05-16

**Authors:** Ayana Hart, Caroline A. Schrodt, Jennifer C. Hunter, David Ham, Elizabeth Soda, Joseph Perz, Kiran Perkins

## Abstract

**Background:** Throughout the COVID-19 pandemic, CDC Division of Healthcare Quality Promotion (DHQP) has provided technical assistance in support of state, tribal, local, and territorial health departments for COVID-19 healthcare outbreak management and infection prevention and control (IPC). We characterized the volume and trends of technical assistance provided during the pandemic to inform the future needs of health departments for COVID-19 healthcare IPC and DHQP resources required to meet these needs. **Methods:** In January 2020, DHQP began receiving COVID-19 IPC TA requests directly from health departments for remote assistance or from CDC staff on field deployments providing onsite support. DHQP subject-matter experts provided responses via e-mail or, for more complex inquiries, outbreaks, or field deployments, via phone consultations. Records of e-mail communications and phone consultations were entered into an inquiry database for tracking. We calculated the number, mean, and range of technical-assistance responses by jurisdiction and by month from January 2020 through December 2021. We designated months as high-volume periods for technical assistance if inquiries surpassed the 75th percentile. **Results:** In total, 1,869 IPC technical-assistance responses were provided. Of all technical-assistance responses, 1,725 (92%) were to state or local health departments, 115 (6%) were tribal nations, and 28 (2%) were US territories. IPC technical assistance was provided to all 50 states and the District of Columbia, 16 tribal nations, and 5 US territories. The average total number of technical assistance responses per site during the 24-month period was 34 to state and local HDs (range, 2–111), 6 to tribal nations (when tribal nation was specified; range, 1–17), and 6 to US territories (range, 1–15). E-mail communications comprised 1,164 responses (62%); phone consultations made up the remaining 705 responses (38%). Of phone consultations, 350 (50%) were with CDC field deployers providing onsite support to health departments. The average number of technical-assistance responses provided each month across all jurisdictions was 78 (range, 0–334); months with high volumes included April–August 2020 and January 2021. **Conclusions:** These findings highlight the high-level collaboration between federal and state, tribal, local, and territorial health department partners in remote and onsite support of COVID-19 prevention and response efforts in healthcare settings. Variations in monthly volumes of health-department COVID-19 healthcare IPC technical assistance requests may reflect factors such as fluctuations in community infection rates and changes in CDC IPC guidance. The ability to provide effective technical assistance during pandemic response depends on the CDC maintaining sufficient healthcare IPC staffing and expertise.

**Funding:** None

**Disclosures:** None

